# Melanoma of the Liver and Gut

**DOI:** 10.1093/jcag/gwaa006

**Published:** 2020-02-20

**Authors:** Jenan Ghaith, Scott Fung, Serra Stefano

**Affiliations:** 1 Department of Medicine, Division of Gastroenterology, University of Toronto, Toronto, Ontario, Canada; 2 University Health Network, Toronto General Hospital, Toronto, Ontario, Canada; 3 Department of Laboratory Medicine and Pathobiology, University of Toronto, Toronto, Ontario, Canada; 4 Department of Gastrointestinal and Pancreas Pathology, University Health Network, Laboratory Medicine Program, Toronto, Ontario, Canada

A 76-year-old Asian male with HBeAg-negative chronic hepatitis B infection, not on antiviral therapy, was referred to the hepatology clinic for investigation of recurrent liver lesions. His past medical history was notable for hypertension, polycystic kidney disease, chronic kidney disease, treated prostate adenocarcinoma and recently exenterated malignant melanoma of the right eyelid. The latter was followed by negative clinical and radiological surveillance confirming the absence of metastases.

There was no personal or family history of colorectal or hepatocellular carcinoma. He also reported no gastrointestinal symptoms, including RUQ abdominal pain, weight loss, gastrointestinal bleeding episodes and symptoms of decompensated liver disease. Blood work at the time of presentation shown a normocytic anemia but normal liver enzyme level and function. Hepatitis B serology is reported in Table 1.

**Table 1.** Hepatitis B serology results

Test	HBs Ag*	HBs Ab^†^	HBe Ag^‡^	HBe Ab^§^	HBV DNA^‖^	Alpha-Fetoprotein
**Result** (units)	Positive	Positive	Negative	Positive	3070 (IU/mL) (Normal: undetectable)	2.0 µg/L (Normal <5.0 µg/L)

*Hepatitis B Surface Antigen.

^†^Hepatitis B Surface Antibody.

^‡^Hepatitis B e Antigen.

^§^Hepatitis B e Antibody.

^‖^Hepatitis B virus DNA.

**Figure 1.**
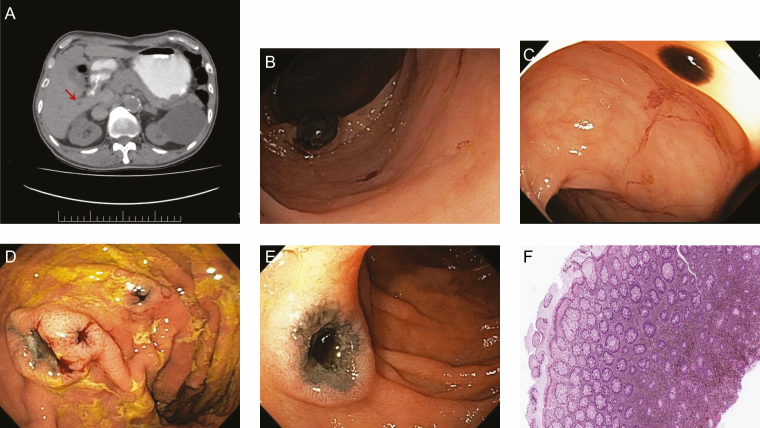
(**A**) Cross-sectional image of the liver with demonstrated hepatic lesion suggestive of malignancy. (**B**) Raised pigmented nodule noted in the large bowel. (**C**) Flat, freckle-like nodule at the right colon. (**D**) Multiple ulcerated pigmented lesion in the stomach body. (**E**) Large ulcer with pigmented base at the duodenum. (**F**) Histological sample demonstrating infiltration of pigment secreting neoplastic cells.

Computed tomography of the abdomen was concerning for new liver lesions but radiographic characteristics were not diagnostic for hepatocellular carcinoma. In view of patient’s age and the possibility of a gastrointestinal primary tumour, endoscopic examination was warranted in order to exclude primary colorectal malignancy (Figure 1A).

Colonoscopy demonstrated multiple nodular and flat lesions with central pigmentation in the ascending colon and throughout the transverse colon (Figure 1B and C). Similarly, upper gastrointestinal endoscopy revealed numerous pigmented gastric and duodenal nodular lesions (Figure 1D and E).

Microscopically, the lamina propria of the stomach, duodenum and colon (Figure 1F) were in infiltrated by neoplastic atypical cells containing cytoplasmic pigment which spared the glands. Immunohistochemistry for S100 highlighted the neoplastic cells, consistent with metastatic melanoma.

Following endoscopy, the patient was started on tenofovir disoproxil fumarate 300 mg daily to suppress chronic HBV infection. He was promptly referred to the Melanoma Clinic and is scheduled to start double immunotherapy (checkpoint inhibition) for metastatic melanoma.

